# Bone mineral density and risk of cardiovascular disease in men and women: the HUNT study

**DOI:** 10.1007/s10654-021-00803-y

**Published:** 2021-09-13

**Authors:** Laxmi Bhatta, Aivaras Cepelis, Sigrid A. Vikjord, Vegard Malmo, Lars E. Laugsand, Håvard Dalen, Arnulf Langhammer, Imre Janszky, Linn B. Strand, Ben M. Brumpton

**Affiliations:** 1 K.G. Jebsen Center for Genetic Epidemiology, Department of Public Health and Nursing, NTNU, Norwegian University of Science and Technology, Trondheim, Norway; 2 Department of Public Health and Nursing, Faculty of Medicine and Health Science, NTNU, Norwegian University of Science and Technology, Trondheim, Norway; 3 Department of Cardiology, St. Olavs Hospital, Trondheim, Norway; 4 Department of Circulation and Medical Imaging, NTNU, Norwegian University of Science and Technology, Trondheim, Norway; 5 Department of Emergency Medicine, St. Olavs Hospital, Trondheim, Norway; 6 Department of Medicine, Levanger Hospital, Nord-Trøndelag Hospital Trust, Levanger, Norway; 7 Cardiac Clinic, St. Olavs Hospital, Trondheim University Hospital, Trondheim, Norway; 8 Department of Neurology, Medical School, University of Pécs, Pécs, Hungary; 9 Department of Medicine, St. Olavs Hospital, Trondheim University Hospital, Trondheim, Norway

**Keywords:** Bone mineral density, Cardiovascular disease, Atrial fibrillation, Myocardial infarction, Ischemic stroke, Hemorrhagic stroke

## Abstract

The association between bone mineral density (BMD) and cardiovascular disease (CVD) is not fully understood. We evaluated BMD as a risk factor for cardiovascular disease and specifically atrial fibrillation (AF), acute myocardial infarction (AMI), ischemic (IS) and hemorrhagic stroke (HS) and heart failure (HF) in men and women. This prospective population cohort utilized data on 22 857 adults from the second and third surveys of the HUNT Study in Norway free from CVD at baseline. BMD was measured using single and dual-energy X-ray absorptiometry in the non-dominant distal forearm and T-score was calculated. Hazard ratios (HR) and 95% confidence intervals (CI) were calculated from adjusted cox proportional hazards models. The analyses were sex-stratified, and models were adjusted for age, age-squared, BMI, physical activity, smoking status, alcohol use, and education level. Additionally, in women, we adjusted for estrogen use and postmenopause. During a mean follow-up of 13.6 ± 5.7 years, 2 928 individuals (12.8%) developed fatal or non-fatal CVD, 1 020 AF (4.5%), 1 172 AMI (5.1%), 1 389 IS (6.1%), 264 HS (1.1%), and 464 HF (2.0%). For every 1 unit decrease in BMD T-score the HR for any CVD was 1.01 (95% CI 0.98 to 1.04) in women and 0.99 (95% CI 0.94 to 1.03) in men. Point estimates for the four cardiovascular outcomes ranged from slightly protective (HR 0.95 for AF in men) to slightly deleterious (HR 1.12 for HS in men). We found no evidence of association of lower distal forearm BMD with CVD, AF, AMI, IS, HS, and HF.

**Supplementary Information:**

The online version contains supplementary material available at 10.1007/s10654-021-00803-y.

## Background

Cardiovascular disease (CVD) is a major public health problem and the main cause of loss of disability-adjusted life years and premature death globally [1, 2]. Bone remodeling is a continuous lifelong process involving removal of mineralized bone (bone resorption) followed by the formation of bone matrix that becomes mineralized (bone formation) [3]. Calcification of the arterial tissue in atherosclerosis seems to be regulated by mechanisms similar to those involved in bone remodeling [4], while decreased bone mineral density (BMD) has been associated with development of atherosclerosis in elderly individuals [5]. Other factors such as oxidative stress, inflammation, free radicals and lipid metabolism are all involved in both bone [6, 7] and cardiovascular health [8]. In addition, increased blood calcium and parathyroid hormone levels and dysfunction of sympathetic nervous system have been indicated in abnormal bone remodeling, low BMD and pathogenesis of atrial fibrillation (AF) [9–12].

Previous studies have shown that stroke [13, 14] or heart failure (HF) [15] predisposes patients to lower BMD, mainly due to physical inactivity. Recently, some epidemiological studies reported prospective associations between low BMD and higher incidence of stroke [16, 17], HF [18, 19], acute myocardial infarction (AMI) [20], and mortality [21, 22]. However, previous studies did not distinguish between ischemic and hemorrhagic stroke [16, 17, 23] that have different etiology and risk factors [24]. Also, to the best of our knowledge, the association with AF has not been previously investigated.

The aim of the present study was to evaluate BMD as a risk factor for any CVD, and specifically AF, AMI, ischemic and hemorrhagic stroke and HF in a large population-based study of men and women. We hypothesized that low BMD is associated with increased AF and atherosclerosis risk including AMI and ischemic stroke, with potential sex differences.

## Methods

### Study design and population

**Fig. 1:**
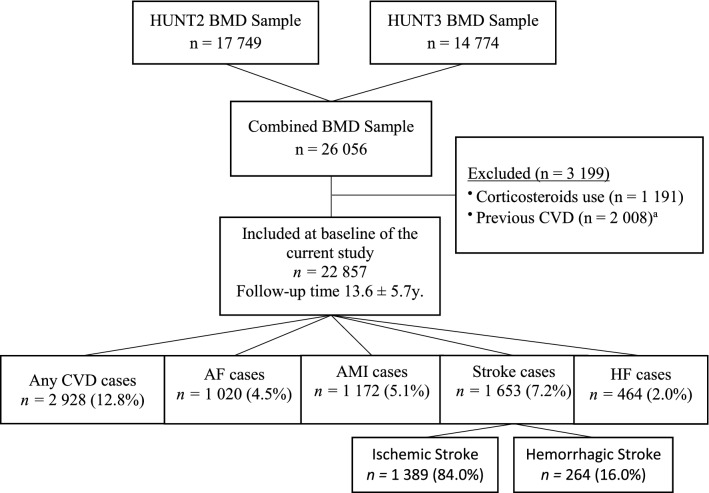
Flowchart of the study population. HUNT (Trøndelag Health Study), BMD (bone mineral density), CVD (cardiovascular disease), AF (atrial fibrillation), AMI (acute myocardial infarction), HF (heart failure). ^a^Including a history of AF, AMI, stroke, and HF

The HUNT Study is the largest Norwegian population-based health study [25]. All adults residing in the northern part of Trøndelag county (n = 94 194 in 1995–1997, n = 93,860 in 2006–2008) were invited to undergo clinical examinations, blood sampling, interviews, and questionnaires in four surveys (HUNT1 1984–86, HUNT2 1995–97,HUNT3 2006–08 and HUNT4 2017–2019) [26]. Bone densitometry was not performed in HUNT1, thus, in the present study, we utilized data from HUNT2 and HUNT3 where a total of 65 215 (69.2% of those invited) and 50 796 (54.1%) individuals participated, respectively. Forearm bone densitometry was performed in 17 749 participants in HUNT2 (21,734 invited; 81.7% participated) and 14 774 participants in HUNT3 (22,490 invited; 65.7% participated) resulting in a total sample of 26 056 adults (Fig. 1). Of the total 26 056 sample, 6 467 individuals (24.8%) participated in both HUNT2 and HUNT3. Participants were selected for bone mineral density measurements in two different ways: 1) a random sample was selected (a 5% random sample from HUNT2 and 10% random sample from HUNT3 among all participants or as part of a 30% random sample from the female birth cohorts or random sample from young-HUNT1 participants) and 2) a symptom sample was selected for the HUNT Lung Study [26], where participants who were asked to participate in spirometry measurements were also asked to participate in bone mineral density measurements. Relevant symptoms included wheezing or breathlessness during the last 12 months at baseline, a history of asthma or ever use of asthma medication. The detailed participant selection process is presented in Figure S1.

Among participants with asthma or COPD we have excluded 1 191 individuals (4.6%) who were current users of inhaled corticosteroids, which could have influenced their BMD measurements [27]. To examine incidence, we excluded subjects with a prior history of cardiovascular diseases including AF, AMI, HF and stroke (n = 2 008, 7.7%), resulting in a total sample of 22,857. Flow chart of participant selection process is illustrated in Fig. 1.

### Bone mineral density

In HUNT2, BMD was measured using single-energy X-ray absorptiometry (SXA) (DTX 100, Osteometer Meditech A/S, Copenhagen, Denmark). Daily calibration of the densitometers was performed with equipment-specific phantoms [28]. Measurements were taken in the non-dominant distal forearm, while the dominant arm was used in the case of previous fractures in the non-dominant arm (2.5% of cases). The distal region was 24 mm proximal to the point at which radius and ulna are 8 mm apart [29]. In HUNT3, BMD was measured using dual-energy X-ray absorptiometry (DTX200, Osteometer Meditech A/S, Copenhagen, Denmark) (n = 9 147) and DTX100 (n = 5 627).

BMD was standardized as T-scores. In both sexes separately, we calculated the T-score as observed BMD minus mean BMD from a reference population divided by standard deviation (SD) of reference population. The reference population was a healthy female population (excluded individuals with self-reported osteoporosis, arthritis, hip, or wrist fractures, hyper- or hypothyroidism and use of corticosteroids) aged 20–39 years from the HUNT Study [22]. Further, BMD T-score was categorized according to the WHO criteria as normal (T-score ≥ – 1.0), osteopenia (– 1.0 to – 2.5), and osteoporosis (≤ – 2.5) [30].

### Cardiovascular disease ascertainment

Incident cases were ascertained by linking the HUNT data with full hospital records in Nord-Trøndelag County from 1995 to 2015. The diagnoses were based on International Statistical Classification of Diseases and Related Health Problems (ICD).

Medical records were manually reviewed by fellow cardiologist and AF was diagnosed based on ICD-10 code I48. The patient was considered as having AF if the electrocardiogram (ECG) could be classified as AF or atrial flutter according to the standard criteria based on the American College of Cardiology consensus guideline [31]. If an ECG scan was not in the digital medical record, the written records were further reviewed for ECG interpretation and, in doubtful cases, the information was evaluated separately by specialist in cardiology and internal medicine, and then discussed in a consensus meeting [32]. In the cases where an ECG was not taken at all, but patients had described irregular heartbeats or periods of fast, irregular pulse, it was not considered AF in our study.

AMI was defined and diagnosed by the caregiving cardiologists and physicians according to the European Society of Cardiology/American College of Cardiology consensus guidelines and consisted of ICD-9 code 410 and ICD-10 codes I21 and I22 [31]. Criteria for AMI included: specific clinical symptoms according to case history information, changes in blood levels of cardiac enzymes, and electrocardiogram changes as defined in the American and European consensus guidelines. If the cardiologists or physicians judged the event to not be a valid AMI, the event was deleted from the registry. A small part of the AMI diagnoses (2%) from medical records have been manually validated [33] and an ongoing validation study (unpublished) found that 92% of the cases (n = 1194) was type 1 AMI.

Ischemic stroke consisted of ICD-9 codes 433 and 434 and ICD-10 code I63 (all positions), while hemorrhagic stroke consisted of ICD-9 codes 430, 431 and 432 and ICD-10 codes I60, I61 and I62. Electronic medical records and diagnostic imaging of hospital admissions for stroke in Norway has been shown to have high sensitivity and positive predictive values in validation studies [34, 35]. HF diagnosis was based on ICD-10 code I50. In addition, we aggregated AMI, any stroke and HF cases into any CVD.

### Covariates

A self-administrated questionnaire was used to assess participants’ smoking status (never, former and current), physical activity (inactive, low, medium and high), alcohol use (abstainers, light, moderate and heavy drinkers), education (< 10, 10–12, > 12 years) and medical history of common chronic diseases. A detailed description of the covariates can be found elsewhere [36]. Body mass index (BMI) was calculated by dividing body weight (kg) by height (m) squared (kg/m^2^). Estrogen users were defined as women reporting current or previous use of systemic estrogen pills or patches, while non-users as women reporting “never use” of estrogen. Postmenopausal women were defined as those who self-reported use of estrogen or answered negative to “Do you still menstruate?” question.

### Statistical analysis

Baseline characteristics were presented using means (SDs) for continuous variables and numbers (percentages) for categorical variables. For the individuals participating at both HUNT2 and HUNT3, HUNT2 was regarded as baseline.

To investigate the prospective association between BMD T-score and outcomes we used Cox proportional hazard models to estimate hazard ratios (HRs) and 95% confidence intervals (CIs). Risk time was calculated from baseline until the first event of interest, death, emigration or end of follow-up (30th November 2015), whichever came first. We used follow-up time as the time scale in our analysis. We tested the proportionality of hazards using log–log curves and Schoenfield's test. We fitted cause-specific models, thus participants with competing events (deaths) were censored at the time of the event. Missing data on covariates were imputed using multiple imputation with chained equations, M = 20.

The non-linearity in the relationship between BMD T-score and outcomes was assessed using the restricted cubic splines with three knots. The number of knots were determined using Akaike (AIC) and Bayesian information criterion (BIC). We observed no deviation from linearity by comparing the Cox proportional hazard models with and without cubic spline terms using Wald test and likelihood ratio tests.

We reviewed the literature and performed a Directed Acyclic Graph analysis to select covariates that could cause both BMD and CVD (Figure S2). A minimally adjusted model included age and age-squared (Model 1). The age-squared term was used to account for the possible non-linearity of age influencing the exposure of interest. Further to this, we controlled for BMI, physical activity, smoking status, alcohol use, and education level (Model 2). We additionally adjusted for estrogen and postmenopause in the analysis among women (Model 3). The E-value was calculated to quantify the strength of residual confounding that would require to explain away the estimates [37]. All analyses were prior stratified by sex due to the sex differences in BMD and bone turnover [38].

We performed the data analyses using Stata 13.1 for Windows 10 (StataCorp). The study received ethics approval from the Regional Committee for Medical Research Ethics (REK 2015/1462). All study participants gave informed written consent.

## Results

A total of 22,857 individuals with BMD data were included in the main analysis. The mean BMD T-score and age were − 1.28 ± 1.82 and 53.25 ± 17.50, respectively for women and 0.002 ± 1.65 and 45.81 ± 15.55, respectively for men. Among women, 3 061 (19.8%) used estrogen and 10,013 (64.7%) were postmenopausal at baseline. Participants categorized as having osteoporosis were more likely to have history of fractures, lower BMI and lower education, be current smokers (men only) and physically inactive (Table 1).

**Table 1:** Characteristics of 22 857 participants stratified by sex

Characteristic	Female (n = 15 484)				All (− 7.91 to 5.84) (n = 7 373)	Male (n = 7 373)		
	All (− 9.20 to 3.79) (n = 15 484)	Distal BMD T-score (in categories)				Distal BMD T-score (in categories)		
		Normal (≥ − 1.0) (n = 7 946)	Osteopenia (− 1.0 to − 2.5) (n = 3 677)	Osteoporosis (≤ − 2.5) (n = 3 861)		Normal (≥ − 1.0) (n = 5 613)	Osteopenia (− 1.0 to − 2.5) (n = 1 346)	Osteoporosis (≤ − 2.5) (n = 414)
At baseline [mean ± SD or n (%)]								
BMD T-score	− 1.28 ± 1.82	0.14 ± 0.80	− 1.68 ± 0.43	− 3.8 ± 0.98	0.002 ± 1.65	0.65 ± 1.21	− 1.58 ± 0.42	− 3.63 ± 1.04
BMD	0.45 ± 0.08	0.51 ± 0.04	0.43 ± 0.03	0.34 ± 0.04	0.59 ± 0.07	0.62 ± 0.05	0.53 ± 0.03	0.44 ± 0.05
Fractures	3 100 (20.0)	972 (12.2)	805 (21.9)	1 323 (34.3)	1 290 (17.5)	913 (16.3)	293 (21.8)	84 (20.3)
*Missing*	623 (4.0)	148 (1.9)	169 (4.6)	306 (7.9)	215 (2.9)	134 (2.4)	41 (3.1)	40 (9.7)
Smoking								
Never	7 443 (48.1)	3 405 (42.8)	1 813 (49.3)	2 225 (57.6)	2 686 (36.4)	2 235 (39.8)	385 (28.6)	66 (15.9)
Former	3 337 (21.5)	1 837 (23.1)	802 (21.8)	698 (18.1)	2 205 (29.9)	1 600 (28.5)	436 (32.4)	169 (40.8)
Current	4 350 (28.1)	2 627 (33.1)	973 (26.5)	750 (19.4)	2 400 (32.6)	1 728 (30.8)	504 (37.4)	168 (40.6)
*Missing*	354 (2.3)	77 (1.0)	89 (2.4)	188 (4.9)	82 (1.1)	50 (0.9)	21 (1.6)	11 (2.7)
Education								
< 10y	6 420 (41.5)	2 186 (27.5)	1 743 (47.4)	2 491 (64.5)	1 940 (26.3)	1 310 (23.3)	433 (32.2)	197 (47.6)
10–12y	5 466 (35.3)	3 676 (46.3)	1 134 (30.8)	656 (17.0)	3 870 (52.5)	3 142 (56.0)	600 (44.6)	128 (30.9)
> 12y	2 632 (17.0)	1 848 (23.3)	576 (15.7)	208 (5.4)	1 349 (18.3)	1 051 (18.7)	260 (19.3)	38 (9.2)
*Missing*	966 (6.2)	236 (2.9)	224 (6.1)	506 (13.1)	214 (2.9)	110 (2.0)	53 (3.9)	51 (12.3)
Physical activity								
Inactive	2 838 (18.3)	1 350 (17.0)	652 (17.7)	836 (21.6)	1 283 (17.4)	958 (17.1)	244 (18.0)	81 (19.6)
Low	4 227 (27.3)	2 268 (28.5)	1 059 (28.8)	900 (23.3)	1 605 (21.8)	1 212 (21.6)	312 (23.2)	81 (19.6)
Medium	5 208 (33.6)	3 973 (37.4)	1 231 (33.5)	1 004 (26.0)	2 489 (33.7)	1 906 (33.9)	456 (33.9)	127 (30.7)
High	849 (5.5)	624 (7.9)	157 (4.3)	68 (1.8)	1 008 (13.7)	826 (14.7)	155 (11.5)	27 (6.5)
*Missing*	2 362 (15.3)	731 (9.2)	578 (15.7)	1 053 (27.3)	988 (13.4)	711 (12.7)	179 (13.3)	98 (23.6)
Alcohol use								
Abstainers	7 532 (48.6)	3 943 (37.0)	1 931 (52.5)	2 658 (68.8)	1 713 (23.2)	1 179 (21.0)	357 (26.5)	177 (42.7)
Light	6 527 (42.2)	4 239 (53.4)	1 430 (38.9)	858 (22.2)	3 708 (50.3)	2 895 (51.6)	656 (48.8)	157 (37.9)
Moderate/Heavy	982 (6.3)	666 (8.4)	209 (5.7)	107 (2.8)	1 822 (24.7)	1 478 (26.3)	298 (22.1)	46 (11.3)
*Missing*	443 (2.9)	98 (1.2)	107 (2.9)	238 (6.2)	130 (1.8)	61 (1.1)	35 (2.6)	34 (8.1)
Age (y)	53.25 ± 17.50	43.04 ± 13.60	56.58 ± 15.52	71.06 ± 8.61	45.81 ± 15.55	42.91 ± 13.62	51.02 ± 16.94	68.12 ± 12.55
BMI (kg/m^2^)	26.71 ± 4.69	26.66 ± 4.86	26.90 ± 4.75	26.64 ± 4.27	26.76 ± 3.83	27.01 ± 3.77	26.10 ± 3.89	25.46 ± 3.98
*Missing*	63 (0.4)	18 (0.2)	14 (0.4)	31 (0.8)	18 (0.2)	7 (0.1)	6 (0.4)	5 (1.2)
Estrogen use^a^	3 061 (19.8)	1 562 (19.7)	882 (24.0)	617 (16.0)	–	–	–	–
*Missing*	4 050 (26.2)	2 048 (25.8)	836 (22.7)	1 166 (30.2)	–	–	–	–
Postmenopause^a^	10 013 (64.7)	3 943 (49.6)	2 804 (76.3)	3 266 (84.6)	–	–	–	–
*Missing*	2 327 (15.0)	1 530 (19.3)	383 (10.4)	414 (10.7)	–	–	–	–
At follow-up [n (%)]								
Any CVD	2 093 (13.5)	500 (6.3)	563 (15.3)	1 030 (26.7)	835 (11.3)	523 (9.3)	214 (15.9)	98 (23.7)
AF	670 (4.3)	211 (2.7)	184 (5.0)	275 (7.1)	350 (4.8)	248 (4.4)	74 (5.5)	28 (6.8)
Ischemic stroke	1 042 (6.7)	232 (2.9)	293 (8.0)	517 (13.4)	347 (4.7)	211 (3.8)	90 (6.7)	46 (11.1)
Hemorrhagic stroke	192 (1.2)	46 (0.6)	51 (1.4)	95 (2.5)	72 (1.0)	41 (0.7)	19 (1.4)	12 (2.9)
AMI	772 (5.0)	196 (2.5)	198 (5.4)	378 (9.8)	400 (5.4)	254 (4.5)	102 (7.6)	44 (10.6)
HF	349 (2.3)	79 (1.0)	93 (2.5)	177 (4.6)	115 (1.6)	72 (1.3)	31 (2.3)	12 (2.9)

SD (standard deviation), BMD (bone mineral density), BMI (body mass index), CVD (cardiovascular disease), AF (atrial fibrillation), AMI (acute myocardial infarction), HF (heart failure)

^a^Percentage expressed among women only

### Association of BMD and cardiovascular disease

Among women free of any CVD events at baseline, there were 2 093 incident cases of CVD (13.5%), 670 AF (4.3%), 772 AMI (5.0%), 1 042 ischemic stroke (6.7%), 192 hemorrhagic stroke (1.2%), and 349 HF (2.3%). Among men free of any CVD events at baseline, there were 835 incident cases of CVD (11.3%), 248 AF (4.8%), 400 AMI (5.4%), 347 ischemic stroke (4.7%), 72 hemorrhagic stroke (1.0%), and 115 HF (1.6%).

We found no evidence for association between 1 unit decrease in distal forearm BMD T-score and CVD, AF, AMI, ischemic stroke, hemorrhagic stroke, and HF in men and women (Table 2). Among women the HRs (95% CI) were 1.01 (0.98–1.04) for CVD, 0.99 (0.94–1.05) for AF, 0.99 (0.94–1.04) for AMI, 1.03 (0.98–1.07) for ischemic stroke, 1.05 (0.95–1.16) for hemorrhagic stroke, and 1.02 (0.94–1.10) for HF (Model 2, Table 2). Similar results were observed in Model 3 (Table 2). Among men the HRs were 0.99 (0.94–1.03) for CVD, 0.95 (0.88–1.02) for AF, 0.96 (0.90–1.03) for AMI, 1.03 (0.96–1.11) for ischemic stroke, 1.12 (0.96–1.32) for hemorrhagic stroke, and 0.97 (0.84–1.11) for HF (Model 2, Table 2).

**Table 2:** Associations between distal forearm bone mineral density T-score and the risk of cardiovascular disease stratified by sex

	Any CVD (n = 2928)	AF (n = 1 020)	AMI (n = 1172)	Ischemic stroke (n = 1389)	Hemorrhagic stroke (n = 264)	HF (n = 464)
Female (n = 15,484)						
No. of cases (%)	2 093 (13.5)	670 (4.3)	772 (5.0)	1 042 (6.7)	192 (1.2)	349 (2.3)
Model 1	0.99 (0.97–1.03)	0.96 (0.92–1.02)	0.98 (0.93–1.03)	1.02 (0.97–1.06)	1.05 (0.96–1.16)	0.99 (0.92–1.07)
Model 2	1.01 (0.98–1.04)	0.99 (0.94–1.05)	0.99 (0.94–1.04)	1.03 (0.98–1.07)	1.05 (0.95–1.16)	1.02 (0.94–1.10)
Model 3	1.01 (0.98–1.04)	0.99 (0.94–1.05)	0.99 (0.94–1.04)	1.03 (0.98–1.07)	1.05 (0.95–1.16)	1.02 (0.94–1.10)
E-value (CI) for Model 3	1.09 (1.00)	1.05 (1.00)	1.12 (1.00)	1.19 (1.00)	1.28 (1.00)	1.15 (1.00)
Male (n = 7 373)						
No. of cases (%)	835 (11.3)	248 (4.8)	400 (5.4)	347 (4.7)	72 (1.0)	115 (1.6)
Model 1	0.99 (0.94–1.03)	0.92 (0.85–0.99)	0.96 (0.90–1.03)	1.02 (0.95–1.10)	1.11 (0.95–1.30)	0.95 (0.83–1.09)
Model 2	0.99 (0.94–1.03)	0.95 (0.88–1.02)	0.96 (0.90–1.03)	1.03 (0.96–1.11)	1.12 (0.96–1.32)	0.97 (0.84–1.11)
E-value (CI) for Model 2	1.13 (1.00)	1.30 (1.00)	1.25 (1.00)	1.21 (1.00)	1.49 (1.00)	1.22 (1.00)

BMD (bone mineral density), BMI (body mass index), CVD (cardiovascular disease), AF (atrial fibrillation), AMI (acute myocardial infarction), HF (heart failure)

Hazard ratios and 95% confidence intervals were derived from Cox proportional hazards models

Model 1 adjusted for age, age-squared

Model 2 adjusted for age, age-squared, BMI, physical activity, smoking status, alcohol use, and education level

Model 3 (female only) adjusted for model 2 and estrogen use and postmenopause

Hazard ratios for 1 unit decrease in distal bone mineral density T-score

## Discussion

In this prospective study including 22 857 adults, there was no evidence of an association between BMD and CVD. Specifically, for each CVD end points, we did observe an indication of a small protective effect on atrial fibrillation and actue myocardial infarction in men, and a small increased risk of hemorrhagic stroke in men, however these associations lacked precision.

There is some previous evidence available for an association between BMD and CVD incidence including myocardial infarction or coronary artery disease [20, 39], stroke [16, 17, 23] and HF [18, 19]. A meta-analysis of prospective studies found an association between low BMD and CVD, and death due to CVD, however after adjustment for publication bias the estimates were attenuated [40]. No large-scale population-based studies have been conducted in Europeans. However, regarding more specific CVD outcomes, one small prospective study observed a modest increased risk of AMI for 1 SD decrease in femoral neck and total hip BMD after adjusting for BMI, age, diabetes, hypertension, smoking and hypertriglyceridemia [20]. Additionally, the Cardiovascular Health Study found lower total hip BMD to be associated with 13% higher HF risk in non-black men, but not women [19].

All previous studies that examined stroke incidence did not distinguish between ischemic and hemorrhagic stroke [16, 17, 23]. Although, in line with previous studies, we found a slightly higher risk of overall stroke for every 1 SD decrease in BMD (HR 1.05, 95% CIs 0.98 to 1.12 in men), the increase was not seen when looking at ischemic stroke specifically. Previously, it has been hypothesized that higher stroke risk is due to increased bone demineralization that leads to vascular calcification and accelerated atherosclerosis [4, 5]. Our study suggests that the overall association with stroke might be due to the hemorrhagic type. This is also supported by the observed null or protective association with acute myocardial infarction that shares common atherosclerotic pathways with ischemic stroke.

Hemorrhagic stroke has different risk factors and etiology than the ischemic type [24]. For example, high blood pressure and alcohol use has a more direct linear relationship with hemorrhagic than ischemic stroke. Also, subarachnoid hemorrhage, a subtype of hemorrhagic stroke, is most caused by a head injury. A previous study found that participants who had experienced a hemorrhagic stroke were at a higher hip/femur fracture risk compared with those who had experienced an ischemic stroke [41]. Poor bone health is a major risk factor for falls, while falls itself is the main cause of fractures and traumatic head injuries [42]. Therefore, there might be a link between low BMD and higher risk of stroke due to head injury. In addition, poor bone health in pre-menopause women could indicate fragility and higher risk of slip and falls, whereas low BMD in postmenopausal is more commonly seen due to hormonal changes [43]. Considering that hemorrhagic stroke is associated with poorer outcomes and 1.5-fold higher mortality than ischemic stroke [44, 45], further studies are needed to confirm our findings and clarify potential mechanisms.

To our knowledge, there has been no earlier studies investigating the association between BMD and atrial fibrillation. Abnormal bone remodeling and increased bone resorption can cause excess release of calcium from the bone mass leading to hypercalcemia [46]. Calcium ions play a major role in cellular electrophysiology and high levels are associated with increased risk of cardiovascular disease [47]. However, blood calcium levels in relation to AF risk is less known and parathyroid hormones with complex calcium regulatory system play a role [48]. In addition, the use of bisphosphonates, a first-line therapeutic agents for treating osteoporosis, have been found to increase AF risk in a randomized clinical controlled trial [49]. However, a meta-analysis of three RCTs and four observational studies did not find a higher risk of AF in bisphosphonate users [50]. In our study, we did not find any evidence for increased AF risk with lower BMD, while serum calcium levels and bisphosphonate use were not assessed.

Our large population-based study had a long follow-up, information on a wide range of confounders, high participation rate and carefully reviewed hospital and register information.

We identify several limitations of this study. Although, total hip dual X-ray absorptiometry (DXA) is the gold standard for bone mineral density measurement with excellent prediction of hip fractures and future osteoporosis, we have used single and dual X-ray absorptiometry of the distal forearm. Forearm was chosen as a measurement site due to practical reasons such as reduced radiation dose especially for women in fertile age, easy and readily standardized assessment, less expensive equipment and shorted duration of the measurement (no undressing needed), all of which makes it more suitable for large-scale population-based studies [51]. Also, forearm measurements have high accuracy of 2% compared to an accuracy error of 8–10% for spine site [52, 53]. Forearm has high accuracy due to the limited amount of surrounding tissue and the precision of bone mass measurements [54]. In addition, forearm BMD measures have been shown to be highly correlated with whole-body BMD and has same accuracy and predictive ability of generalized osteoporotic bone loss at any site [51, 55]. Also, previous studies showed that forearm BMD is a good predictor of future fractures at any site in women [56] and men [57]. Lastly, by utilizing the non-dominant forearm we reduced potential residual confounding by leisure and work-related physical activity [54]. Overall, forearm is a valid site in assessing whole body BMD and fractures risk within the population.

Secondly, DTX100 (SXA) was used in HUNT2 and DTX200 (DXA) in HUNT3. The agreement between them has been found to be acceptable by a previous validation study within HUNT sample, which found that DXA measured slightly higher BMD value than the SXA with the mean difference of 4.5% per g/cm^2^ (unpublished). Also, a previous study in Norway found that Root Mean Square Standard Deviation (RMS SD) for SXA and DXA forearm was 4.6 (4.2–5.1) and 6.8 (6.1–7.4), respectively, and the corresponding coefficients of variation was 1.0% and 1.4% [58].

The third limitation is that stroke and AMI cases have been ascertained through hospital recorded ICD codes but, unlike atrial fibrillation, not all cases were manually validated. However, validation studies of stroke and AMI from electronic medical records in Norway reported high sensitivity and positive predictive values [33–35]. Nevertheless, lack of manual review and no validation studies for heart failure diagnosis is a major limitation in this study. In addition, observational studies are generally susceptible to confounding. However, for residual confounding to be influencing our results considerably a potential confounder would have to be strongly associated with both BMD and the outcome and be unrelated to the confounders already included in our models. Finally, for each association we calculated the E value [37], which supported that remaining residual confounding was unlikely to influence our results.

## Conclusion

Our findings contribute to the knowledge of bone health and cardiovascular disease. We found no evidence of risk of cardiovascular diseases in women and men with lower distal forearm bone mineral density. Although we did not observe statistically significant associations between BMD and cardiovascular outcomes, our point estimates of hazard ratios may be compatible with a small protective effect on atrial fibrillation and actue myocardial infarction in men, and a small increased risk of hemorrhagic stroke in men. Future studies are needed to replicate these findings.

## Supplementary Information

Below is the link to the electronic supplementary material.

Supplementary file1 (DOCX 68 kb)
