# Supplementary material for: Bone mineral density and risk of cardiovascular disease in men and women: the HUNT study
Source: Eur J Epidemiol. 2021 Sep 13;36(11):1169–77. doi: 10.1007/s10654-021-00803-y (PMC8629874; doi:10.1007/s10654-021-00803-y)
Supplement: Supplementary file 1 — Supplementary file1 (DOCX 68 kb) [file 10654_2021_803_MOESM1_ESM.docx]

Supplementary Material:

**Bone Mineral Density and Risk of Cardiovascular Disease in Men and Women: the HUNT Study**

Laxmi Bhatta^1*^, Aivaras Cepelis^2^, Sigrid A Vikjord^2^, Vegard Malmo^3 4^, Lars E Laugsand^5^, Håvard Dalen^5 6 7^, Arnulf Langhammer^2^, Imre Janszky^2 8^, Linn B Strand^2^, Ben M Brumpton ^1 9*^

***Corresponding authors:** K.G. Jebsen Center for Genetic Epidemiology, Department of Public Health and Nursing, Faculty of Medicine and Health Science, NTNU, Norwegian University of Science and Technology, Trondheim, Postbox 8905, NO-7491 Trondheim, Norway.

Laxmi Bhatta: [laxmi.bhatta@ntnu.no](mailto:laxmi.bhatta@ntnu.no)

Ben Brumpton: ben.brumpton@ntnu.no

Invited to HUNT2

n = 94 194

Lung Study invited

(symptom ^a^ sample)

n = 8 544 (13.1%)

Random Female Birth Cohorts invited

n = 9 855 (15.1%)

Participated

n = 6 720 (78.7%)

Random sample invited

n = 3 335 (5.1%)

Participated

n = 8 079 (82.0%)

Participated

n = 2 950 (88.5%)

Participated

n = 65 215 (69.2%)

Valid distal forearm BMD measurement n = 17 749 (81.7%)

Invited to HUNT3

n = 93 860

Lung Study (symptom ^c^ + spirometry participants^d^) invited

n = 5 879 (11.6%)

Random Female Birth Cohorts

n = 3 573 (7.0%)

Participated

n = 3 959 (67.3%)

Random sample larger municipal.

n = 3 354 (6.6%)

Participated

n = 664 (55.6%)

Participated

n = 2 408 (67.4%)

Participated

n = 2 116 (63.1%)

Random sample Young HUNT1^b^

n = 1 195 (2.4%)

Participated

n = 50 796 (54.1%)

Valid distal forearm BMD measurement n = 14 774 (65.7%)

DTX200

DTX100

Small municipalities ^e^ invited

n = 8 489 (16.7%)

Participated

n = 5 627 (66.3%)

**Figure S1**. Flowchart of BMD sample selection

^a^ Answering 'yes' to asthma symptoms, diagnosis or medication use.

^b^ The Young-HUNT Survey is the adolescent part of the HUNT Study, including participants aged 13-19 years conducted in 1995-1997

^c^ included participants who reported attacks of wheezing or breathlessness during the last 12 months, a history of asthma or to ever have used asthma medication at baseline.

^d^ 5% random sample and asthma symptom sample.

^e^ includes HUNT2 bone densitometry participants, HUNT3 asthma symptom, 5% random sample

Age

Sex

Body mass index

Education

Physical activity

BMD CVD

Alcohol use

Smoking status

Estrogen use (Females only)

Postmenopause (Females only)

**Figure S2.** Directed Acyclic Graph for multivariable model
